# 间变性淋巴瘤激酶阳性大B细胞淋巴瘤误诊为多发性骨髓瘤1例报告并文献复习

**DOI:** 10.3760/cma.j.cn121090-20250118-00033

**Published:** 2025-06

**Authors:** 智慧 李, 波 王, 鑫 吕

**Affiliations:** 1 临沂市中心医院血液科，临沂 276400 Department of Hematology, Linyi Central Hospital, Linyi 276400, China; 2 临沂市中心医院病理科，临沂 276400 Department of Pathology, Linyi Central Hospital, Linyi 276400, China

## Abstract

间变性淋巴瘤激酶阳性大B细胞淋巴瘤（ALK^+^ LBCL）临床罕见，其浆母细胞分化、结外骨及骨髓受累表现少见，患者常规化疗敏感性低、预后差。本文回顾性分析临沂市中心医院收治的1例以骨质破坏及骨髓受累为主要表现被误诊为多发性骨髓瘤的ALK^+^ LBCL患者的诊治过程并进行相关文献复习。患者为64岁男性，因咳嗽、咳痰伴胸背部疼痛半月入院，被诊断为多发性骨髓瘤，接受VRD方案化疗3个疗程后被评估为部分缓解。患者自行停药2个月后出现截瘫，经胸椎内占位切除病理诊断ALK^+^ LBCL，给予ALK抑制剂联合CHOP方案化疗3个疗程，加用达雷妥尤单抗2个疗程后短暂缓解。

## Abstract (en)

Anaplastic lymphoma kinase-positive large B-cell lymphoma（ALK^+^ LBCL）is rarely encountered in clinical practice. Some cases have rare plasmoblastic differentiation, extranodal bone and bone marrow involvement, low sensitivity to conventional chemotherapy, and poor prognosis. This study retrospectively analyzed the diagnosis and treatment of a patient with ALK^+^ LBCL who was first misdiagnosed with multiple myeloma based on the presence of bone destruction and bone marrow involvement in Linyi Central Hospital, and the relevant literature was reviewed. The patient was a 64-year-old man who was admitted to the hospital for half a month with cough and sputum accompanied by chest and back pain. He was diagnosed with multiple myeloma and was partially relieved by three courses of VRD chemotherapy. Two months after the self-discontinuation of these medications, he experienced paraplegia. Pathological examination following the resection of an intrathoracic space-occupying lesion confirmed ALK-positive large B-cell lymphoma. The patient was treated with three cycles of ALK inhibitor combined with the CHOP regimen, followed by two additional cycles of daratumumab, which led to transient clinical remission.

间变性淋巴瘤激酶阳性大B细胞淋巴瘤（ALK^+^ LBCL）是一种罕见的CD20阴性侵袭性淋巴瘤，占弥漫大B细胞淋巴瘤比例不足1％，各年龄阶段均可发病，但通常发生在免疫功能正常的年轻患者，中位年龄35岁，男女比例为3.5∶1。大多数患者表现为颈部或纵隔淋巴结肿大，可有结外累及，诊断时多为进展期，预后差，5年生存率仅为28％～34％[1]–[2]。本病临床罕见，肿瘤细胞具有浆母细胞/免疫母细胞形态及浆细胞表型，导致其与浆细胞肿瘤鉴别困难。本文报道1例误诊为多发性骨髓瘤的ALK^+^ LBCL患者的诊疗过程并进行相关文献复习。

## 病例资料

患者，男，64岁，因“咳嗽、咳痰伴胸背部疼痛半月”于2023年8月31日入住我院呼吸科。查体：浅表淋巴结未触及肿大，胸骨无压痛，肝脾肋下未及。血常规：WBC 5.16×10^9^/L、HGB 120 g/L、PLT 304×10^9^/L、红细胞沉降率78 mm/h。生化：总蛋白112.40 g/L、白蛋白31.5 g/L、球蛋白80.9 g/L、肌酐75 µmol/L、钙2.43 mmol/L、LDH 1 163 U/L。胸部CT示多发肋骨、椎体及肩胛骨骨质破坏。考虑多发性骨髓瘤转入血液科，完善骨髓穿刺：骨髓肿瘤细胞占24％，破裙样、多核及巨大肿瘤细胞均可见，形态呈病理性畸形。诊断意见：多发性骨髓瘤。流式细胞术检测：3.97％（占有核细胞）的细胞表达CD138、CD38、CD56、胞质（c）Lambda，不表达CD20、CD19、cKappa，为恶性单克隆浆细胞。骨髓病理：浆细胞呈大片状、弥漫性分布，细胞胞体偏大，胞核呈圆形或不规则形、常偏位，胞质丰富。免疫组化：CD20、CD3、Kappa阴性；CD38、CD138、Lambda弥漫阳性。意见：多发性骨髓瘤（肿瘤细胞约占90％）。FISH：RB1（13q14）缺失阳性；（1q21/1p32）CKS1B扩增阳性；t（4；14）IGH/FGFR3阳性；t（11；14）IGH/CCND1阴性；t（14；20）IGH/MAFB阴性；t（14；16）IGH/MAF阴性；（17p13）TP53/CEP17阴性。骨髓染色体：46,XY[10]。血β_2_微球蛋白3.34 mg/L。血免疫球蛋白（Ig）G 72 g/L，血清免疫固定电泳IgG-λ型。血清蛋白电泳：单克隆免疫球蛋白（M蛋白）占比48.1％，M蛋白54.06 g/L。血清游离轻链：κ 7.45 mg/L、λ 532.00 mg/L，λ/κ比值为71.4。尿本周蛋白电泳阳性，类型为λ游离轻链型。尿蛋白电泳定量：M蛋白占比58％，M蛋白104.98 mg/24 h。颈椎、胸椎、腰椎MRI平扫：诸椎体及附件区多发局灶性骨质破坏，部分胸椎旁软组织影（大者1.7 cm×0.8 cm）。患者拒绝PET-CT检查。根据以上检查结果诊断患者为多发性骨髓瘤［IgG-λ型、DS分期Ⅲ期A、国际分期系统（ISS）分期Ⅱ期、修订后的ISS（R-ISS）分期Ⅱ期、R2-ISS分期Ⅲ期，RB1缺失阳性、CKS1B扩增阳性、IGH/FGFR3阳性、LDH升高、骨旁髓外浆细胞瘤，梅奥骨髓瘤分层及风险调整治疗分层系统（mSMART3.0）高危］。给予VRD方案（硼替佐米2 mg，第1、4、8、11天；来那度胺25 mg，第1天至第21天；地塞米松20 mg，第1、2、4、5、8、9、11、12天）化疗3个疗程，评估疗效为部分缓解（PR），之后患者未再就诊。

2024年1月23日患者因“双下肢活动障碍2 d”来我院就诊，查体：T2～T5水平棘突压痛、叩击痛，自乳头以下水平躯干、肛门周围及会阴部、双下肢皮肤感觉消失，双上肢肌力4级、双下肢肌力1级。血常规：WBC 4.75×10^9^/L、HGB 127 g/L、PLT 340×10^9^/L、红细胞沉降率56 mm/h。生化：总蛋白68.1 g/L、白蛋白33.3 g/L、球蛋白34.8 g/L、肌酐38 µmol/L、钙2.03 mmol/L、LDH 573 U/L。EB病毒阴性。血IgG 23.2 g/L、血β_2_微球蛋白1.91 mg/L。血清蛋白电泳：M蛋白占比21.6％，M蛋白14.70 g/L。血清免疫固定电泳：IgG-λ型。血清游离轻链：κ 10.00 mg/L、λ 123.00 mg/L，λ/κ比值为12.3。尿蛋白电泳定量：M蛋白占比25.3％，M蛋白28.84 mg/24 h。尿本周蛋白电泳阳性，类型为λ游离轻链型。颈椎、胸椎、腰椎MRI平扫：诸椎体及附件区异常信号，符合多发性骨髓瘤MRI表现，T2、T3椎体椎管内占位病变。患者于2024年1月29日接受胸椎后路椎管减压植骨融合内固定术，术后病理（胸椎管）送检组织：病变细胞体积较大、异型明显，可见大片坏死，胞质较丰富，核仁明显，部分区域核偏位，少部分区域见R-S样大细胞，形态符合淋巴造血系统恶性肿瘤，结合免疫组化，符合ALK^+^ LBCL伴浆母细胞分化。免疫组化：肿瘤细胞CK、MUM1、Lambda、ALK、Bcl-2、EMA、Oct2、CD138、CD38、LCA、CD4均阳性；CD30、EBER、c-Myc、CD20、CD56、CD117、CD79a、CD3、CyclinD1、Kappa、Bcl-6、BOB1均阴性；CD10部分阳性、Ki-67约90％阳性、P53约5％阳性。PET-CT：全身多发骨^18^F-氟代脱氧葡萄糖（^18^F-FDG）摄取增高灶，SUVmax：18.05，较大病灶位于L4椎体区，大小约54 mm×43 mm×22 mm，边界欠清，局部见软组织肿块形成，SUVmax：9.33。纵隔血池：SUVmax：1.35；肝血池：SUVmax：1.84。予初诊骨髓活检加做免疫组化显示ALK大片阳性（图1）。IGH基因重排阳性。CARS-ALK融合基因阳性。综上诊断ALK^+^ LBCL（Ann Arbor分期Ⅳ期A；IPI评分5分、高危；NCCN-IPI评分7分、高危）。应用ALK抑制剂联合CHOP方案化疗（克唑替尼250 mg，每日1次；环磷酰胺1.2 g，第1天；吡柔比星70 mg，第1天；长春新碱2 mg，第1天；泼尼松100 mg，第1天至第5天）3个疗程，联合达雷妥尤单抗800 mg化疗2个疗程。患者短暂缓解，后期放弃治疗，于2024年12月死亡。

**图1:**
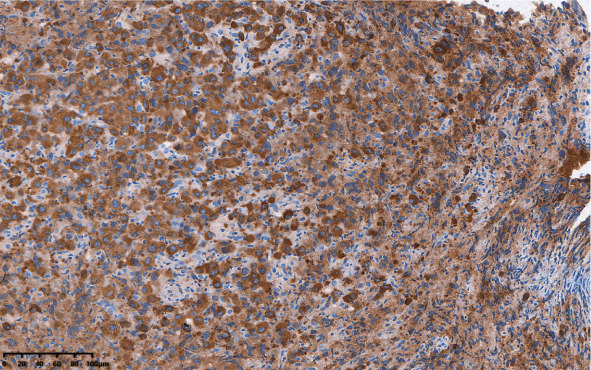
患者骨髓活检ALK免疫组化结果（×100）

## 讨论及文献复习

ALK^+^ LBCL是一种罕见的CD20阴性侵袭性淋巴瘤，预后差。1997年Delsol等[3]首次报道，2022年WHO造血和淋巴组织肿瘤分类[4]将其定义为具有浆细胞表型的ALK阳性单形性大免疫母细胞样B细胞的侵袭性肿瘤。本病具有特征性的ALK重排及形态学、免疫表型特征，通常发生在免疫功能正常的年轻患者，大多数表现为颈部或纵隔淋巴结肿大，可有结外累及包括舌、鼻咽、胃、肝、脾、骨、软组织和皮肤等部位，25％病例出现骨髓浸润[5]。诊断时多为进展期（60％），IPI评分和Ann Arbor分期是重要的预后因素[2]，IPI高危组患者预后差，总体5年生存率28％～34％[1]–[2]，中位生存期为1.83年。Ⅰ～Ⅱ期患者的2年和5年总生存（OS）率（分别为76％和66％）高于Ⅲ～Ⅳ期患者（2年OS率：27％；5年OS率：8％）。

ALK^+^ LBCL虽有特征性ALK表达，但因其罕见性及其形态学可呈浆母细胞样分化，与其他具有浆母细胞分化的肿瘤均表达浆细胞标志物如CD38、CD138、MUM-1且特征性强表达EMA，而CD20及其他B细胞和T细胞标志物阴性，临床易误诊。结合本病例的诊治过程，ALK^+^ LBCL需要与以下疾病鉴别[3],[5]–[7]：①浆细胞瘤/骨髓瘤：以浆细胞、浆母细胞恶性增殖为特征，出现一系列器官功能异常，临床表现为骨痛/骨折、肾功能异常、贫血、高钙血症等，可出现脊椎、皮肤、软组织等髓外浸润，但淋巴结累及少见[8]。ALK^+^ LBCL与浆细胞瘤/骨髓瘤的肿瘤细胞均表达浆细胞标志物，实验室检查可见IGH重排、M蛋白升高（Ig类型主要为IgA、IgG[2]）、骨及骨髓浸润等表现，此类表现易与浆细胞瘤/骨髓瘤临床表现重叠从而发生误诊。ALK^+^ LBCL与浆细胞瘤/骨髓瘤鉴别重点为ALK表达，目前最常见的细胞遗传学改变[1]是t（2;17）（p23;q23），从而产生CLTC-ALK融合蛋白，而浆细胞瘤/骨髓瘤无ALK融合蛋白的表达。本例患者骨髓活检结果显示出与胸椎组织免疫组化ALK表达的一致性，提示肿瘤细胞为同源。同时，CARS-ALK融合基因阳性也支持了ALK^+^ LBCL的诊断，尽管此融合基因相关文献报道少见，但其仍可能作为ALK^+^ LBCL的驱动基因，导致ALK持续活化，从而发挥致癌作用。需要注意的是，多发性骨髓瘤高水平LDH患者比例较低[9]，而本例患者初诊时LDH>1 000 U/L，应警惕淋巴瘤的可能。综上，对于浆细胞瘤/骨髓瘤的病例要重视浆细胞形态及其抗原表达情况，应充分考虑有无伴浆细胞分化淋巴瘤可能，建议骨髓病理中常规加做ALK表达检测，避免漏诊或误诊；对于进展迅速如出现椎体压迫、髓外进展的病例，要重视重新组织活检的必要性。②浆母细胞淋巴瘤（PBL）：CD20阴性非霍奇金淋巴瘤，肿瘤好发于结外如口腔、胃肠道，HIV、EB病毒感染者多见，Ki-67指数常>90％。其肿瘤细胞呈浆母细胞形态，表达浆细胞标志物，与本病浆母细胞分化时一致，ALK、人类疱疹病毒8型（HHV8）阴性是与本病的鉴别点。

ALK^+^ LBCL对常规化疗敏感性差[6]，加入新的靶向药物ALK抑制剂如克唑替尼可获得短期改善，而获得性克唑替尼耐药的分子发生机制以及新一代ALK抑制剂阿来替尼和洛拉替尼如何克服这种耐药性仍在探索中[7]。本例患者肿瘤细胞在形态学及免疫表型上与多发性骨髓瘤相似，理论上针对肿瘤细胞表达浆细胞标志物CD38、CD138可以应用蛋白酶体抑制剂或CD38单克隆抗体进行治疗，而且在PBL中证实，联合应用硼替佐米、来那度胺、达雷妥尤单抗可以提高疗效[10]。本例患者前期应用硼替佐米、来那度胺达到PR疗效，后期借鉴PBL治疗经验联合达雷妥尤单抗取得一定疗效，侧面提示此类患者可尝试应用。然而，本患者初诊存在骨、软组织、骨髓浸润，处于疾病进展期，IPI评分高，预后极差，即使联合靶向药物其生存期也仅为16个月。

综上，本例ALK^+^ LBCL患者以肿瘤细胞致骨质破坏、骨髓浸润症状为首发表现罕见，预后差，一线选择ALK抑制剂联合CHOP方案不佳的情况下，本例患者联合达雷妥尤单抗可获得短暂缓解，为提高治疗效果提供了新的思路，但由于是个案报道，仍需进行大样本量的研究以便深入了解、诊断和治疗疾病。
